# Targeting the cyclin-dependent kinases (CDK) 4/6 in estrogen receptor-positive breast cancers

**DOI:** 10.1186/s13058-015-0661-5

**Published:** 2016-02-09

**Authors:** Richard S. Finn, Alexey Aleshin, Dennis J. Slamon

**Affiliations:** Geffen School of Medicine at UCLA, Department of Medicine, Division of Hematology Oncology, 2825 Santa Monica Blvd, Santa Monica, CA 90404 USA; Stanford School of Medicine, 291 Campus Drive, Stanford, CA 94305 USA

## Abstract

Despite significant advances in early detection and treatment, breast cancer still remains a major cause of morbidity and mortality for women. Our understanding of the molecular heterogeneity of the disease has significantly expanded over the past decade and the role of cell cycle signaling in both breast cancer oncogenesis and anti-estrogen resistance has gained increasing attention. The mammalian cell cycle is driven by a complex interplay between cyclins and their associated cyclin-dependent kinase (CDK) partners, and dysregulation of this process is one of the hallmarks of cancer. Despite this, initial results with broadly acting CDK inhibitors were largely disappointing. However, recent preclinical and phase I/II clinical studies using a novel, oral, reversible CDK4/6 inhibitor, palbociclib (PD-0332991), have validated the role of CDK4/6 as a potential target in estrogen receptor-positive (ER+) breast cancers. This review highlights our current understanding of CDK signaling in both normal and malignant breast tissues, with special attention placed on recent clinical advances in inhibition of CDK4/6 in ER+ disease.

## Background

Breast cancer is a global disease, with a yearly incidence of over 1.3 million, accounting for over 23 % of all malignancies [1]. Our knowledge of the molecular diversity and drivers of specific subtypes of breast cancer has paved the way for the rational design and clinical development of targeted agents. These are designed to increase efficacy while sparing many of the traditional toxicities associated with chemotherapy and the success of this approach has been clearly demonstrated by the development of anti-estrogens and HER2-targeted agents for hormone receptor-positive and HER2-amplified breast cancers, respectively. Despite these advances in our treatment armamentarium, many patients still develop resistance to both targeted and non-targeted therapeutics, ultimately developing fatal disease and underscoring the need for new therapeutic approaches.

Using temperature-sensitive yeast mutants, Lee Hartwell first identified cell division cycle (CDC) genes as key regulators of cell division some 40 years ago [2]. Paul Nurse subsequently found the human homologues to these genes and named the family cyclin-dependent kinases (CDKs) [3]. In the early 1980s Tim Hunt discovered cyclin molecules in his studies of sea urchin egg division [4]. These molecules were named on the basis of their cyclical appearance and were found to play an important role in binding and activating CDK proteins. This critical array of activators and kinases is now known to be central in regulating cell division and these important accomplishments were recognized by the 2001 Noble Prize in Physiology and Medicine. Today the cell cycle is viewed as an orderly progression of distinct phases (G1, S, G2, M), with various cyclin/CDK combinations being essential in regulating this process. Pursuant to these pivotal observations, multiple studies have linked alterations in cell cycle biology to cancer. In breast cancer, alterations in several cell cycle regulatory proteins have been described, including various cyclins, CDKs, and the *RB* gene product (pRb) [5–7]. Evidence indicates that dysregulation of the cyclin D1:CDK4/6 axis has a role in breast cancer, with some tumors overexpressing cyclin D1 [5]. Additionally, while not necessary for normal mammary gland development, CDK4 and cyclin D1 are required for induction of breast malignancies in mouse models, suggesting that CDK4 inhibition may inhibit breast cancer cells while sparing healthy tissues [6, 7]. The above data seemed to suggest that pharmacological inhibition of the cyclin D1:CDK4/6 axis in cancers may be both efficacious and relatively non-toxic. However, the initial clinical experience with broad specificity, first-generation CDK inhibitors proved to be disappointing, yielding poor efficacy and significant toxicity and raising the question of whether these agents failed due to poor phamacologic characteristics and/or specificities of the compounds or a less essential role of CDK signaling in cancer. Additionally, lack of appropriate patient selection and/or lack of predictive markers of response may have also contributed to these initial clinical failures. Recently, the development of more specific CDK inhibitors has renewed interest in targeting the cell cycle as a novel therapeutic approach in cancer. In a series of preclinical studies using cell line models of human breast cancers, we demonstrated significant growth inhibitory activity of palbociclib (PD-0332991), which is a highly selective inhibitor of CDK4/6 [8]. These observations were followed by a logical translation of the laboratory findings into a phase I/II clinical study that has now demonstrated significant clinical activity in patients with advanced estrogen receptor-positive (ER+) breast cancer [9].

In this review, we further describe the role of cyclin:CDK activity in regulating the cell cycle and focus on the central role of cyclin D:CDK4/6 activity in both normal and malignant tissues. Finally, we discuss the preclinical and clinical experience with CDK inhibitors with particular emphasis on selective CDK4/6 inhibitors.

## Role of CDK4/6 in cell cycle control

The basic regulatory framework of the cell cycle has been extensively investigated and reported in the literature. It is more extensively reviewed elsewhere [10, 11] but a brief summary of these important prior findings follows.

**Fig. 1:**
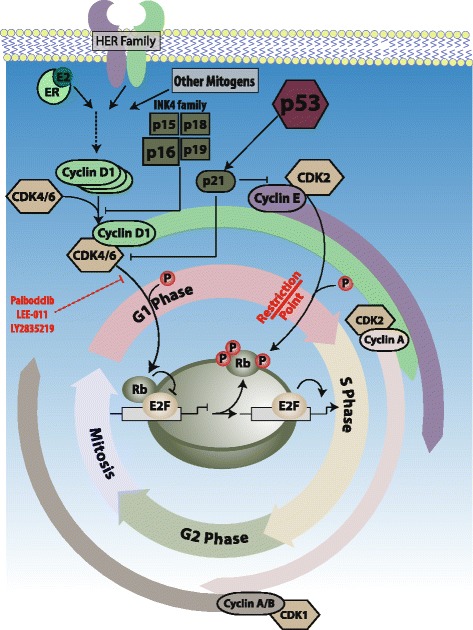
The cyclin D/cyclin-dependent kinase (*CDK*)4/6/retinoblastoma (*Rb*) Pathway and the cell cycle. The mammalian cell cycle is tightly regulated. In the context of breast cancer, both steroid and peptide growth factors drive proliferation through cyclin D/CDK4/6 activation. This results in the hyper-phosporylation of pRb as G1 progresses. When retinoblastoma protein (pRb) is hyper-phosphorylated, the transcription factor E2F is released and the cell cycle progresses through S phase. Small molecule kinase inhibitors of CDK4/6 aim to block the hyper-phosphorylation of pRb inducing a G1 arrest and preventing proliferation. *ER* estrogen receptor

The mammalian cell cycle is classically partitioned into four distinct phases, termed G1, S, G2, and M. An orderly progression between these phases is tightly controlled at 'checkpoints' by the interplay of various cyclins and their associated CDKs [12] (Fig. 1). At least 12 separate genetic loci are known to code for the CDKs and belong to a well conserved family of serine/threonine protein kinases. This family includes three interphase CDKs (CDK2, CDK4, CDK6), one mitotic CDK (CDK1, previously known as CDC2), and a number of regulatory CDKs, such as CDK7, a component of the CDK-activating complex, and transcriptional CDKs (CDK8, CDK9) [11–13]. Unlike CDKs, cyclins are an extremely diverse family of proteins, subdivided into four classes (A-, B-, D-, E-type cyclins) that act as regulatory subunits of the CDK-cyclin holoenzyme [11]. Despite the large number of CDKs and cyclins, only a few have been strongly implicated in breast cancer pathogenesis. This review focuses primarily on CDK4 and CDK6, which have largely overlapping though not entirely identical specificity, as well as cyclin D1, the most characterized member of the D-type (D1, D2, D3) cyclin family [14].

Typically, repression of cell cycle progression is maintained via sequestration of the E2F family of transcription factors by the retinoblastoma gene product (pRb), and other so-called pocket proteins, including p107 and p130 [15]. Upon entering the cell cycle, however, quiescent cells synthesize cyclin D1 in response to specific mitogenic and adhesion signals. Newly synthesized cyclin D1 goes on to form activating complexes with CDK4/CDK6, which then initiate phosphorylation of pRb. The process of phosphorylation mediated by the cyclin D1:CDK4/6 complex lifts pRb’s transcriptional repression of E2F, resulting in transcription of S-phase-specific target genes. One of these genes encodes cyclin E, which associates with CDK2 and further phosphorylates pRb as well as other key mediators of the G1/S checkpoint. This process sets up a positive feedback loop committing cells to irreversibly undergo the G1–S transition (the so called 'restriction point') and to continue the cell cycle in a mitogen-independent manner [16, 17]. In addition to pRb phosphorylation, recent studies have implicated cyclin D:CDK4 as directly acting on pathways involved in proliferation, migration, and response to DNA damage [18–21] by phosphorylating targets such as SMAD2, Cdt1, MARCKS, FOXM1, and PRMT5–MEP50 complex [18, 21–24].

In late S phase CDK2 is further activated by cyclin A2, enabling transition from S phase to G2 phase. Lastly, CDK1 is activated by A-type and B-type cyclins to facilitate the onset and progression of the actual process of mitosis [11].

## Negative regulators of CDK4/6 signaling

CDK4/6 activity is negatively regulated by two families of cyclin kinase inhibitors (CKIs), the INK4 (p16, p15, p18, p19) and CIP/KIP (p21, p27, p57) protein families [11, 25]. These inhibitors, while largely undetectable in cycling cells, are rapidly upregulated in response to inhibitory signals, including transforming growth factor-β, contact inhibition, or senescence [26, 27]. The effect of the CIP/KIP family on the cell cycle machinery is complex and can be both activating and inhibitory under different circumstances [10]. Interestingly, tamoxifen is known to upregulate p21 as well as p27 and the loss of these cell cycle inhibitors has been implicated in anti-estrogen resistance [28]. Within the INK4 (inhibitors of CDK4) family of proteins, p16 seems to be most directly implicated in the pathogenesis of many malignancies and when bound to CDK4/6 abrogates the ability of cyclin D1 to bind effectively, thereby triggering a G1 cell cycle arrest [29]. Additionally, p16 has been implicated in activation of cellular senescence defined as a stable and long-term loss of proliferative capacity and is another process that is frequently dysregulated in cancer [30].

## Non-catalytic functions of the cyclin D:CDK4/6 pathway

Not all effects of the cyclin D:CDK4/6 pathway are driven by phosphorylation, and a non-catalytic role of cyclin D1 is being increasingly recognized. Cyclin D1 is now also implicated in transcriptional regulation of many genes by acting directly at promoter regions and regulating histone acetylation and methylation [31–33]. Cyclin D1 has been shown to interact with ER-alpha, enhancing its activity, while inhibiting the activity of androgen receptor (AR), thyroid hormone receptor-β and peroxisome proliferator activated receptor-γ (PPARγ) [34]. Another well described non-catalytic function of cyclin D1 is the sequestration of p21 and p27, leading to CDK4/6-independent effects on migration and the DNA damage response [35, 36]. The relative degrees to which these non-catalytic functions of cyclin D1 are physiologically relevant in the cell cycle specifically and regulation of cell division and motility are still being determined but they should be considered when evaluating the effects of inhibition of CDK4/6-mediated signaling.

## Role of CDK4/6 in normal development

Given the inherent linearity of CDK–cyclin activation during the cell cycle, it was long believed that loss of an individual CDK would have deleterious effects on cellular proliferation and embryonic development. This notion has been called into question by separate mouse knockouts of CDK2, CDK3, CDK4, and CDK6, all of which are viable [37–40]. However, double knockouts of CDK4 and CDK6 and triple knockouts of cyclin D1, D2, and D3 develop largely normally but die in mid/late gestation from severe anemia and heart abnormalities, respectively. Embryonic fibroblasts from these mice essentially proliferate normally, despite an increased mitogen requirement and slower S phase entry, but they also display less propensity for oncogenic transformation [41, 42]. These data indicate that CDK4 and CDK6 activity may be dispensable in some developmental and normal cellular functions, suggesting that targeted inhibition could be relatively well tolerated by normal tissues.

## Role of CDK4/6 in breast cancer pathogenesis

Alterations in the mechanisms governing the cell cycle are considered a 'hallmark of cancer' and result in uncontrolled cellular proliferation [43]. Numerous lines of evidence point to an important role of a dysregulated cyclin D1:CDK4/6 complex in both the initiation and progression of many cancers, including breast cancer. Dysregulation of the cyclin D1:CDK4/6 axis appears to be an early step in breast cancer pathogenesis given that 'overexpression' of cyclin D1 is frequently found as early as ductal carcinoma in situ and maintained in metastatic lesions but is absent in the earliest lesions such as atypical ductal hyperplasias [44, 45]. The D-type cyclins are known to be dispensable during mammary gland development, but are required for efficient tumor initiation as evidenced by the fact that mice lacking functional cyclin D1 are resistant to cancers initiated by ErbB-2/HER2/*neu* and ras oncogenes, while cyclin D3 null animals are refractory to Notch1-driven T cell acute lymphoblastic leukemia [7, 46, 47]. Additionally, it appears that cyclin D1 and D3 can compensate for one another in driving tumor initiation and progression [48]. Similarly, CDK4 expression appears to be required for ErbB-2 tumorigenesis, but is dispensable for wnt-induced oncogenesis [49]. Further evidence for their role in malignant pathogenesis derives from studies demonstrating that the cyclin D1:CDK4/6 axis is critical for breast cancer maintenance and progression. This is based on data showing ErbB2-driven tumor arrest and senescence in vivo in response to acute cyclin D1 ablation or targeted inhibition of CDK4/6 [47].

While cyclin D1:CDK4/6 complexes have a central role in regulating the initiation of the cell cycle, activating mutations in CDK4/6 are exceedingly rare in cancer. Nevertheless, amplification of CDK4 and cyclin D1 have been reported in upwards of 15–25 % of breast cancers, while overexpression of cyclin D1 has been reported to occur in over half of all breast cancers in some published studies [44, 45, 50, 51]. The recent Cancer Genome Atlas publication presented data from 510 tumor specimens from 507 patients for which a comprehensive genomic analysis was performed [52]. When analyzed by intrinsic subtype of breast cancer, alterations in cell cycle genes varied, with cyclin D1 amplification being found most frequently in the luminal A, B and HER2 enriched subtypes at frequencies of 29 %, 58 %, and 38 %, respectively. Conversely, amplification of cyclin E1 was more common in the basal subtype. Similar to cyclin D1, gains in CDK4 were more common in the luminal A, B and HER2 enriched subgroups: 14 %, 25 %, and 24 %, respectively. Additional alterations that would be hypothesized to antagonize CDK4/6 dependence, such as lower pRb expression or *RB* loss/mutation, were common in the basal type as well (20 % for mutation/loss).

Amplification of both cyclin D1 and CDK4 is especially high in luminal B (58 % and 25 %, respectively) and HER2-expressing subtypes (38 % and 24 %, respectively), intermediate in luminal A (29 % and 14 %, respectively), and lower in basal-like tumors that tend to also have frequent loss of pRb [52]. In retrospect, other alterations that would antagonize CDK4/6 dependence, such as lower *RB* expression or *RB* loss/mutation, are more common in the basal subtype as well.

## Interplay of CDK4/6 and endocrine signaling in breast cancer

The cross-talk between peptide growth factor and steroid hormone signaling has been an area of active research in breast cancer and a focus of clinical research studies. ER and HER2 signaling appear to be putative 'drivers' in the biology of about 60 % and 20–25 % of breast cancers, respectively [53]. While the therapeutic approaches to these subtypes focus on these respective receptors, the two pathways potentially converge, ultimately exerting their downstream effects on the cyclin D:CDK4/6 pathway.

ER+ breast cancers are largely dependent on estrogen signaling for proliferation and survival [54], with ER inhibition leading to reduced tumor cell viability and cell cycle arrest in the G1 phase [55, 56]. ER signaling is known to upregulate cyclin D1 levels and potentiate multiple signaling pathways largely culminating in upregulation of CDK4/6 activity [57, 58]. Not surprisingly, hormone-based therapeutic strategies form the backbone of treatment of ER+ breast cancers. However, not all ER+ cancers respond to this approach and, among those that do, acquired resistance is not uncommon. Data indicate that this may be mediated, at least in some of these cancers, by deregulation of multiple alternative mitogenic pathways (for example, HER2, PI3K/AKT, and so on) that can potentiate cyclin D1:CDK4/6 signaling in an ER-independent fashion. Also, as mentioned above, cyclin D1 can independently activate ER and a majority of cyclin D1 overexpressing breast cancers are ER+ [51, 59]. These findings suggest a potential role for cyclin D:CDK4/6-mediated signaling in the estrogen independence of ER+ breast cancers [60].

## Therapeutic targeting of the cyclin D:CDK4/6 pathway

Cell cycle regulation has been identified as an attractive target for targeted drug therapy. Given their kinase activity, the CDKs were pursued as drug targets. A large number of drug discovery programs have yielded potent small molecule CDK inhibitors, with several compounds successfully entering preclinical and early clinical trials. Until relatively recently, however, many CDK inhibitors have shown poor clinical activity accompanied by an undesirable adverse event profile. In general, CDK inhibitors can be broken down into two classes: first-generation inhibitors such as flavopiridol, R-roscovitine, and UCN-01, which tended to be less specific and broad in their ability to block a number of CDKs (pan-CDK inhibitors); and second-generation agents that are more specific to certain CDKs. The latter group of compounds has now shown more potent activity against their targets and a more favorable safety profile.

### The first-generation CDK inhibitors

As mentioned, most of the first-generation compounds are not specific for any single CDK enzyme and act primarily as pan-CDK inhibitors. Despite initial enthusiasm generated by preclinical studies, however, many of these compounds suffered from low activity and/or toxicity in clinical studies.

Flavopiridol (National Cancer Institute) is the most studied of all first-generation CDK inhibitors, and is a classic pan-CDK inhibitor. In phase I and II studies, flavopiridol showed minimal single agent efficacy and was associated with several toxicities more typical of traditional cytotoxic agents, including infusion site irritation, gastrointestinal toxicity, and severe neutropenia [61]. In metastatic breast cancers in particular, flavopiridol generated unacceptably high rates of neutropenia [62]. At least a portion of this toxicity is attributable to the inhibition of transcription by the compounds effects on CDK9 and possibly CDK7 that lead to depletion of short-lived cell cycle and anti-apoptotic mRNA transcripts [63]. Though this likely contributes to the in vitro efficacy of flavopiridol on tumors dependent on the expression of such transcripts, off-target effects in healthy tissues would contribute to the severe anti-proliferative toxicity observed in multiple clinical trials of this compound [64].

Other examples of pan-CDK inhibitors include UCN-01 and R-Rescovitine (seliciclib; Cyclacel). UCN-01 is a staurosporine analog with broad activity against CDKs, AKT, Chk1, and protein kinase C. This drug showed good G1/S phase cell cycle arrest, induction of p21 and hypophosphorylation of pRb in preclinical models but phase I studies showed several dose-limiting toxicities, including hyperglycemia, arrhythmia, and pulmonary dysfunction [65, 66]. Results of phase II studies in breast cancer were unimpressive [67].

### Second-generation CDK inhibitors

As mentioned, until recently, CDK inhibitors have shown largely disappointing results in terms of clinical efficacy, safety, and tolerability. One of the main issues associated with first-generation inhibitors is the low specificity toward the target kinases, which can explain their unpredictable and serious side effect profiles. Additionally, some of these agents suffered from suboptimal dosing schedules, typically focusing on intravenous bolus administration that may be insufficient for many solid tumor types that have doubling times in the order of days. Based on these observations, so-called second-generation CDK inhibitors were developed in the late 1990s and early 2000s that showed preferential inhibition of specific CDK subtypes. Initial efforts focused mainly on CDK2 inhibition, given the availability of X-ray crystallographic structures of CDK2 (CDK4 has subsequently been crystalized) [68].

### Specific CDK4/6 inhibitors

Recently, a number of inhibitors specific for CDK4 and CDK6 have entered clinical testing (Table 1). Palbociclib (PD 0332991; Pfizer) is furthest along in clinical development, having received US Food and Drug Administration (FDA) approval on 3 February 2015 for the first-line treatment of advanced post-menopausal ER+, HER2-negative breast cancer in combination with letrozole. It is an orally bioavailable, potent CDK4/6 inhibitor with an in vitro kinase IC50 of 0.01 μM and high selectivity when evaluating 36 other kinases including CDK2 (IC50 > 5 μM) [69]. Preclinical studies have shown that palbociclib behaves very much like an agent specifically targeting CDK4/6. It exhibits potent inhibition of tumor cell proliferation accompanied by a pure G1 arrest, and dephosphorylation of pRb as well as a decrease in E2F-dependent gene expression [70]. Further evidence of palbociclib's targeted design is the fact that it is completely inactive in pRb-negative tumor cell lines and xenografts [9, 60, 70]. In phase I clinical studies palbociclib showed excellent bioavailability with a generally mild to moderate adverse events profile with the major dose limiting toxicities being related mainly to myelosuppression [71].

**Table 1:** Current CDK4/6 inhibitors in clinical development

Compound	Manufacturer	Phase	CDK4/6 in vitro inhibition profile (IC50, nM)
Palbociclib (PD-0332991)	Pfizer, Inc.	Approved	CDK4 (cyclinD1): 11
			CDK4 (cyclinD3): 9
			CDK6 (cyclinD2): 15
Ribociclib (LEE011)	Novartis	III	CDK4 (cyclinD1): 10
			CDK6 (cyclinD2): 40
Abemaciclib (LY2835219)	Eli Lilly	III	CDK4 (cyclinD1): 2
			CDK6 (cyclinD1): 9.9

Using an unbiased screening approach we performed preclinical work aimed at identifying breast cancers that might be growth inhibited by palbociclib and predictive markers of drug response. This was done by evaluating palbociclib’s growth inhibition effects in a large panel of molecularly characterized human breast cancer cell lines. This study identified that cell lines representing either the luminal, ER+ or HER2-amplified subtypes were most sensitive to palbociclib inhibition while those representing the non-luminal subtypes were most resistant [9]. This work also demonstrated consistent synergistic growth inhibitory activity between palbociclib and tamoxifen or trastuzumab in ER+ and HER2-amplified cell models, respectively. Lastly, the drug showed activity in a model of acquired tamoxifen resistance leading to the concept that it may be clinically active in hormone-resistant, ER+ breast cancers.

These data were used to support the clinical development of palbociclib in a phase I/II study of frontline treatment of advanced ER+ post-menopausal breast cancer with a combination of palbociclib and letrozole. The phase I portion enrolled 12 patients and was designed to evaluate the safety of a dosing regimen consisting of 125 mg palbociclib orally given daily on a 3-week on/1-week off regimen in combination with daily letrozole [72]. There were no treatment-related serious adverse events and the most common treatment emergent adverse events were leukopenia, neutropenia, and fatigue. However, there were no instances of neutropenic fever and there were no dose–dose interactions between palbociclib and letrozole.

The phase II study was developed as an open label trial in post-menopausal women with advanced ER+, frontline metastatic breast cancer. It was designed to compare progression-free survival (PFS) as its primary endpoint with safety and overall survival as secondary endpoints and randomized patients to receive either letrozole alone or the combination of letrozole and palbociclib. The study consisted of two parts that enrolled sequentially: part 1 required that patient tumors be ER+, the sole biomarker for study entry; part 2 enrolled the same population but patient tumors were also required to have either CCND1 (cyclin D1) amplification by fluorescence in situ hybridization (FISH) or CDKN2A (p16) loss by FISH as selection biomarkers in addition to the ER+ biomarker. While the preclinical data did not suggest that these genomic markers were required for augmented response, part 2 of the study was designed to determine whether the presence of these biomarkers might further enrich the responsive patient population.

Results from part 1 were presented at the IMPAKT meeting in 2012 [73]. About half the women in each arm had not received any prior neoadjuvant or adjuvant systemic treatment for their diagnosis but about a third had received prior anti-estrogen therapy in early breast cancer settings. There was a significant improvement in PFS in part 1 with the median PFS increasing from 5.7 months with letrozole alone to over 18 months with the combination, resulting in a hazard ratio (HR) of 0.35 (95 % confidence interval (CI) 0.17–0.72, *P* = 0.06). In addition, in patients with measurable disease the response rate increased from 32 to 52 % and the clinical benefit rate increased from 47 to 76 %. Dose reductions and delays were common in the palbociclib arm, but again, the most common treatment-related adverse events were leukopenia, neutropenia, and fatigue, although no instances of neutropenic fevers were reported. Retrospective biomarker analysis for CCND1 amplification and p16 loss was performed in the 66 patients from part 1. Though the groups were small, the HRs for each group demonstrated a consistent benefit regardless of the presence or absence of these biomarkers; biomarkers present (n = 21) HR = 0.37 (95 % CI 0.10–1.40, *P* = 0.13), biomarkers absent (n = 25) HR = 0.19 (95 % CI 0.05–0.67, *P* < 0.01), biomarker unknown (n = 20) HR = 0.59 (95 % CI 0.11–3.08, *P* = 0.53). These data support the preclinical observation that ER positivity may be the best selection biomarker for patients likely to benefit from CDK4/6 inhibition.

An interim analysis combining parts 1 and 2, based on 50 % of events of the 114 needed for the final PFS analysis, was presented at the 2012 San Antonio Breast Cancer Symposium and the final results have been published [73, 74]. These analyses included 165 patients and confirmed the benefit and safety profile observed initially in part 1. Specifically, the final results demonstrated that median PFS increased from 10.2 months with letrozole alone to 20.2 months with the combination (HR = 0.488 (95 % CI 0.319–0.748, *P* < 0.001)). In cohort 1, median PFS was 5.7 months in the letrozole alone arm and was 26.1 months in the combination arm; in cohort 2 these numbers were 11.1 months and 18.1 months, respectively. HRs for both cohorts, 0.299 for cohort 1 and 0.508 for cohort 2, confirmed a benefit regardless of the presence of cyclin D1 amplification or p16, suggesting the most important determinant for benefit in this study is being ER+. The objective response rate for patients with measurable disease was increased from 39 to 54 % with the addition of palbociclib and the clinical benefit rate (complete response, partial response, and stable disease >6 months) for the intent-to-treat population improved from 58 to 81 %. The adverse event profile remained essentially the same. While the incidence of grade 3 and 4 neutropenia was 48 % and 6 %, respectively, there were no cases of neutropenic complications (that is, febrile neutropenia or serious infections). The lack of serious complications from the neutropenia may be explained by the cytostatic effect of CDK4/6 inhibition on the bone marrow which, compared with cytotoxic chemotherapy, results in a relatively short period of neutropenia. In addition, no mucositis or skin toxicity was associated with palbociclib, which are often considered sources of infection with chemotherapy-associated neutropenia. Preclinical studies suggest that CDK4/6 inhibition induces a reversible pharmacologic quiescence in hematopoietic stem/progenitor cells that differs significantly from cytotoxic effects and may explain the clinical observation [75].

Together, the safety and efficacy data from this study resulted in palbociclib receiving a 'Breakthrough Therapy' designation from the US FDA and more recently accelerated approval for advanced ER+ breast cancer [76, 77]. A phase III, double-blind, placebo-controlled study designed to confirm the phase II observations has completed accrual and results are awaited (PALOMA-2/TRIO-22, NCT01740427). Results of the PALOMA-3 study have recently been published and again demonstrate a significant improvement in PFS when palbociclib is used in combination with endocrine therapy [78]. In this large phase III, placebo-controlled, double-blind study, palbociclib and fulvestrant was compared to fulvestrant and placebo. The study demonstrated a doubling of PFS. The PFS in the treatment arm was 9.2 months (95 % CI 7.5–not estimable) compared to 3.8 months (95 % CI 3.5–5.5) in the control arm. Unlike the PALOMA-1/TRIO18 and PALOMA-2/TRIO22 studies, this population of patients had a more endocrine-resistant disease, with the requirement to have progressed on or within 1 month of prior aromatase inhibitor for advanced disease, or within 12 months of completion or discontinuation of therapy for adjuvant therapy. This study also allowed pre-menopausal women that received goserelin as well. The safety profile looked very similar to what was seen in the PALOMA-1/TRIO18 study.

Single-agent activity of palbociclib has also been evaluated in a single arm phase II trial of palbociclib in advanced, heavily pre-treated breast cancer [79]. Despite being tested in a heavily pre-treated cohort of patients (median lines of therapy = 3), single-agent activity was noted (clinical benefit 21 %, stable disease >6 months 14 %). Importantly, as the preclinical data suggested, this activity was seen in women with ER+ or HER2-amplified breast cancers. Myelosuppression again was the most frequently observed adverse event, with 46 % of patients requiring dose reductions and 25 % requiring dose interruptions.

In addition to palbociclib, two other small molecule CDK4/6 inhibitors are currently in early clinical development. Both have had their development programs expedited, going from phase I to phase III based on the palbociclib experience. The molecules and ongoing trials in breast cancer are highlighted in Tables 1 and 2, respectively. Phase I data with LY2835219 (abemaciclib; Eli Lilly) in patients with advanced malignancies was presented at the ASCO 2013 meeting [80]. In this dose escalation study it was determined that the doses in the expansion phase were to be 150 mg and 200 mg twice a day continuously, without a dosing break like with palbociclib. They concluded that it had an acceptable safety profile and early signals of clinical efficacy were seen. Data on an expansion cohort of advanced breast cancer patients have been presented as well [81, 82]. Two cohorts were examined, one with single-agent abemaciclib and one with abemaciclib and fulvestrant for ER+ disease. In the single-agent cohort, 47 patients with all subtypes of breast cancer were enrolled, but significant single-agent activity was seen only in women with ER+ breast cancer. The median lines of prior therapy in this group were 7 (2–16). The overall response rate in the 36 patients with ER+ disease was 33 % and the disease control rate was 80.6 %. Median PFS was 8.8 months for the ER+ cohort compared with 1.1 months in the ER-negative group. In the combination cohort, patients with ER+ metastatic breast cancer (n = 18) were treated with the combination abemaciclib plus fulvestrant. Patients received abemaciclib at 200 mg orally every 12 hours on a continuous schedule. Patients also received 500 mg fulvestrant intramuscularly every month. Patients in this cohort had a median of four lines of prior therapy. The disease control rate in the latter cohort was 72.2 %. Like palbociclib, neutropenia was seen in 40 % of all-grade cases, and 21 % of grade 3/4 cases. There was 66 % all-grades diarrhea reported, of which there were only 6 % grade 3 cases and no grade 4 cases. This side effect seems to indicate some differences between palbociclib and abemaciclib. The dose in phase III breast cancer studies is 150 mg daily every 12 hours, continuously.

**Table 2:** Currently registered clinical studies with CDK4/6 inhibitors in breast cancer

Compound	Setting	Trial primary endpoint	Combination	N	Phase	ClinicalTrials.gov identifier
Palbociclib (PD-0332991)	First-line metastatic	PFS	Letrozole	450	III	NCT01740427 (PALOMA-2)
	Metastatic	PFS	Fulvestrant	417	III	NCT01942135 (PALOMA-3)
	High-risk adjuvant	iDFI	Anti-hormonal	800	III	NCT01864746 (PENELOPE-B)
	Neo-adjuvant	pCR	Anastrozole	29	II	NCT01723774
	Pre-operative	ORR	Letrozole	45	II	NCT01709370
	Metastatic	MTD	Paclitaxel	20	I	NCT01320592
	Neo-adjuvant	Biomarker cCR	Letrozole	306	II	NCT02296801 (PALLET)
	Metastatic	PFS	Exemestane versus capecitabine	348	III	NCT02028507 (PEARL)
	Adjuvant	Treatment discontinuation	Letrozole	160	II	NCT02028507
	Metastatic	Dose/toxicity	TDM-1	17	Ib	NCT01976169
	Neoadjuvant	RCB	Letrozole versus FEC-3	132	II	NCT02400569 (NeoPAL)
	Adjuvant	iDFS	SAT	4600	III	NCT02513394 (PALLAS)
Ribociclib (LEE011)	First-line metastatic	PFS	Letrozole	450	III	NCT01958021
	Pre-surgical	PD	Letrozole	120	II	NCT01919229 (MONALEESA-1)
	Metastatic	DLT, PFS	BYL719, letrozole	300	I/II	NCT01872260
	Metastatic	DLT, PFS	Exemestane, everolimus	185	Ib/II	NCT01857193
	Metastatic	PFS	Letrozole	650	III	NCT01958021 (MONALEESA-2)
	Metastatic	DLT	Letrozole, buparlisib	13	I	NCT02154776
	Metastatic (pre-menopausal)	PFS	Tamoxifen, NSAI	660	III	NCT2278120 (MONALEESA-7)
	Metastatic	DLT/PFS	BYL719^a^ or BKM120	216	I/II	NCT01872260
	Metastatic	PFS	Fulvestrant	660	III	NCT02422615 (MONALEESA-3)
Abemaciclib (LY2835219)	Neoadjuvant	Biomarker	Anastrozole	220	II	NCT02441946 (NeoMONARCH)
	Brain metastasis	Response	Single agent	120	II	NCT02308020
	Metastatic	PFS	NSAI	450	III	NCT02246621 (MONARCH-3)
	Metastatic	Response	Single agent	128	II	NCT021024490 (MONARCH-1)
	Metastatic	PFS	Fulvestrant	630	III	NCT02107703 (MONARCH-2)
	Metastatic	PFS	NSAI, tamoxifen, exemestane, everolimus, trastuzumab	102	I	NCT02057133

^a^BYL719 (Novartis) is a phosphoinositide 3-kinase α-specific inhibitor. *cCR* clinical complete response, *DLT* dose limiting toxicity, *FEC* 5-fluorouracil, epirubicin, cyclophosphamide; *iDFS* invasive disease-free interval, *MTD* maximum tolerated dose, *NSAI* non-steroidal aromatase inhibitor, *ORR* objective response rate, *pCR* pathologic complete response rate, *PD* pharmacodynamic, *PFS* progression-free survival, *RCB* residual cancer burden, *SAT* standard adjuvant therapy, *TDM-1* ado-trastuzumab emtansine

Like palbociclib, LEE011 (ribociclib; Novartis) is being dosed at 600 mg daily, 3 weeks on and 1 week off. Limited data in breast cancer have been presented. In a large phase I study of advanced pRb + solid tumors, single-agent activity was seen in patients with breast cancer [83]. The most common grade 3/4 toxicities at the recommended dose for expansion were neutropenia (26 %), leukopenia (16 %), and lymphonepnia (16 %). LEE011 is now moving ahead into more advanced studies in breast and other cancers. In addition, it is being evaluated in combination with the p110α-specific phosphoinositide 3-kinase inhibitor alpelisib (BYL719) and letrozole and in combination with everolimus plus exemestane. More mature data with both these compounds are eagerly awaited.

## Conclusion

The translational road to effectively targeting the cell cycle has been a long journey from basic science studies to eventual clinical testing. The challenges to this process have been highlighted, and include the identification of the most relevant biologic targets, the development of effective, clinical grade inhibitors of those targets, and ultimately the identification of the appropriate target population to pursue for clinical development. The preclinical observation that palbociclib (PD-0332991) had preferential activity in cell line models that represented the ER+ as well as HER2-amplified subgroups has led to very promising phase II efficacy/safety data in ER+ breast cancers. While single-agent activity has been reported in heavily pre-treated patients with these subtypes, the combination data with letrozole in the first-line treatment of post-menopausal breast cancer has brought the fundamental biology of the cyclin:CDK:RB signaling complex to the forefront of new therapeutic approaches to cancers. At this time, several CDK4/6 inhibitors are moving through clinical development and there will be further research into optimal combinations with other molecularly targeted agents and in other breast cancer settings. The opportunity to target CDK4/6 in HER2-amplified breast cancer remains a very rational goal given the biology and preclinical data demonstrating synergy with trastuzumab [8]. Ongoing and planned tissue acquisition studies will further inform these development strategies. In addition, as further experience is gained, identification of any mechanisms of resistance to CDK4/6 inhibition that may be identified will be of significance in further understanding this pathway and how to improve our therapeutic approach to it. Given the interplay between the steroid hormone and peptide growth factor signaling pathways and their intersection with CDK biology, it is quite likely that our understanding of resistance to CDK4/6 inhibitors will broaden our understanding of the underlying biology of these signaling pathways. Ultimately, if validated in breast cancer, we would hypothesize that, given appropriate selection biomarkers, subgroups of patients with other tumor types may benefit from CDK4/6 inhibition.

## Note

This article is part of a series on ‘*Recent advances in breast cancer treatment’*, edited by Jenny Chang. Other articles in this series can be found at http://breast-cancer-research.com/series/treatment.

## Abbreviations

CDK: Cyclin-dependent kinase
CI: Confidence interval
ER: Estrogen receptor
FDA: Food and Drug Administration
FISH: Fluorescence in situ hybridization
HR: Hazard ratio
PFS: Progression-free survival
pRb: Retinoblastoma protein
RB: Retinoblastoma
